# Stingray Sting Injury to the Hand: A Report of a Rare Case

**DOI:** 10.7759/cureus.82252

**Published:** 2025-04-14

**Authors:** Mohamad Shawal Sjahrial, Lim Chia Hua, Elaine Soh, Shalimar Abdullah, Jamari Sapuan

**Affiliations:** 1 Department of Orthopaedics and Traumatology, Faculty of Medicine, Universiti Kebangsaan Malaysia, Kuala Lumpur, MYS

**Keywords:** envenomation, infection prophylaxis, stingray injury, surgical exploration, wound management

## Abstract

Stingray injuries, though rare, can cause significant complications due to mechanical trauma, envenomation, and secondary infections. This case report describes a stingray injury to the right hand of an aquatic enthusiast and pet shop owner while handling a stingray in his aquarium. The patient arrived at the emergency department with severe, localized pain, a classic sign of stingray envenomation. A comprehensive evaluation, including species identification through a photograph provided by the patient, enabled a targeted treatment plan. Initial management involved thorough wound irrigation, immersion in hot water for pain relief, and surgical exploration to remove any foreign bodies. Prophylactic antibiotics were also administered to reduce the risk of infection. This case emphasizes the need for prompt and thorough management of stingray injuries, even in controlled settings, to prevent complications and optimize outcomes.

## Introduction

Stingrays are cartilaginous fish belonging to the order Myliobatiformes, which includes eight families, such as Potamotrygonidae, commonly known as river stingrays. These species are predominantly found in tropical and subtropical coastal marine environments worldwide. A distinguishing anatomical feature of stingrays is the barb or spine on their tail, which functions as a defensive weapon capable of delivering venom upon contact [1]. The spine is coated with a toxic mucous substance, and when it punctures the skin, venom is introduced into the body, triggering localized physiological responses. Typically, this results in mechanical trauma such as lacerations, muscle and ligament tears, and, in severe cases, bone fractures if the injury penetrates deeply. These injuries increase the risk of secondary infections, including osteomyelitis [2-4]. The severity of injuries caused by stingray spines depends on the species, as the toxicity and size of the spines vary considerably [5]. Furthermore, the venom promotes tissue necrosis, elicits inflammatory responses, and causes intense pain. Its bioactive components include enzymes that induce hemolysis, compounds that stimulate histamine release leading to vasodilation and heightened pain, and activators of the complement system, which exacerbate systemic effects [1].

Clinical symptoms following a stingray injury may appear immediately, ranging from acute local pain to delayed reactions that manifest up to 12 hours post-envenomation [2]. In some instances, long-term complications can develop within 24 hours [4]. Prompt medical attention is crucial to reduce the risk of severe outcomes, such as local or systemic infections, progressive tissue necrosis, allergic reactions to the venom, and chronic pain [5].

The primary goals in managing stingray injuries are to relieve pain, prevent infection, and minimize tissue damage. One essential therapeutic measure is immersing the affected limb in hot water, which helps denature the venom proteins and provides significant pain relief [1]. Analgesics should also be administered to control pain. The wound must be thoroughly irrigated with clean water to remove venom residue and foreign debris, and prophylactic antibiotics should be prescribed to lower the risk of infection [3].

Injuries to the hand are particularly alarming due to its complex anatomy, the potential for deep tissue involvement, and its crucial functional role [3]. We present a case involving a stingray injury to the hand of an aquatic enthusiast and pet shop owner who was handling a stingray in his aquarium. This case highlights that stingray injuries can occur even outside the creature’s natural habitat.

## Case presentation

**Figure 1:**
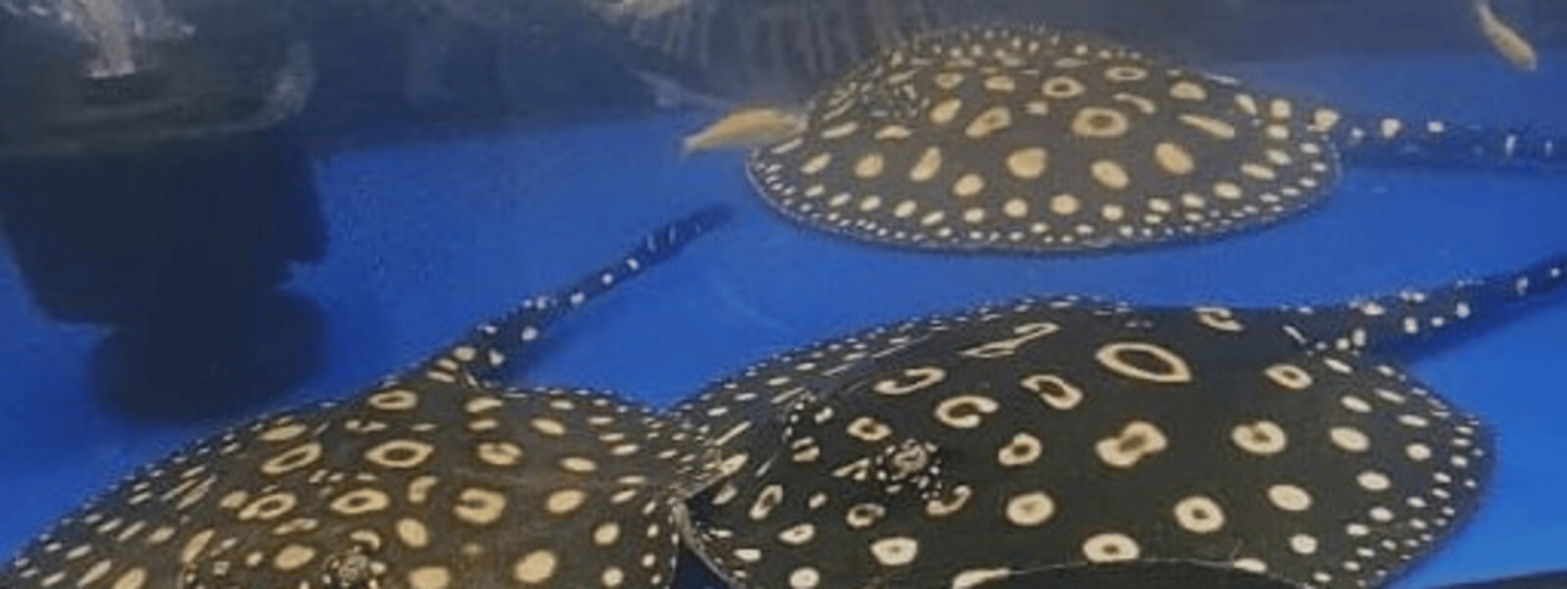
Photograph of the stingray in the aquarium, taken by the patient, that caused the hand injury. The distinctive spotted or mottled pattern with contrasting light spots on its body is characteristic of stingrays belonging to the Potamotrygonidae family.

A 51-year-old male aquatic pet shop owner with no known medical conditions or allergies presented with a wound on his right hand after being stung by a stingray during aquarium cleaning (Figure 1). The injury, located on the radial aspect of his right hand, was accompanied by an electric-like, radiating pain extending proximally to his right shoulder, with a reported pain score of 6 out of 10. The onset of symptoms occurred immediately after being stung by the stingrays. No numbness in the right upper limb and no active bleeding were observed.

**Figure 2:**
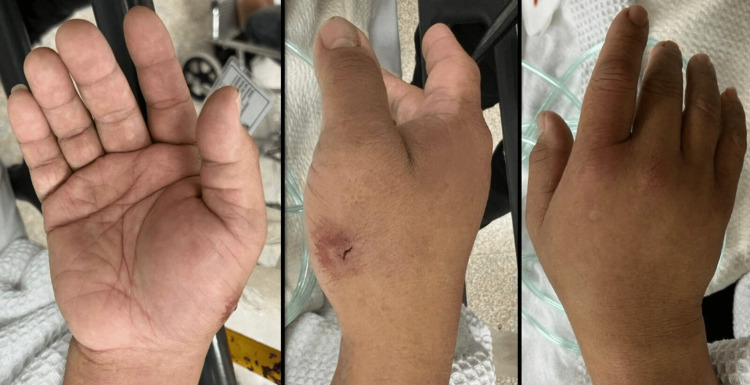
Clinical pictures of the patient’s volar, radial lateral and dorsal aspect of right hand. The wound is located at radiopalmar of the right hand.

**Figure 3:**
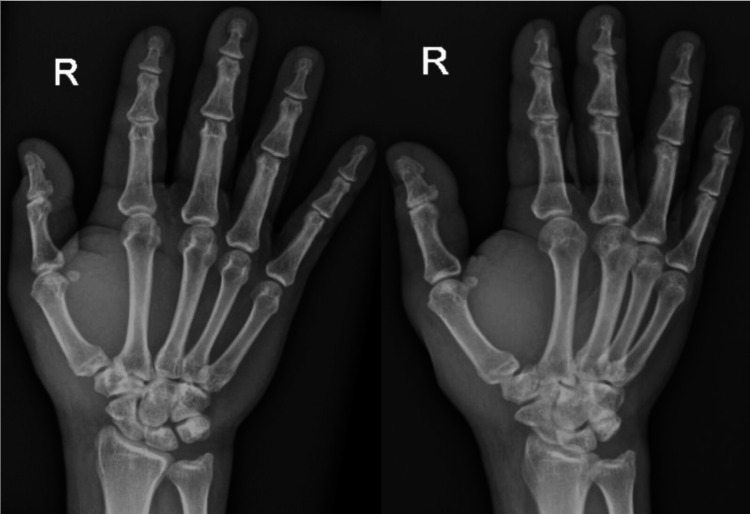
Radiograph of the patient’s right hand in anteroposterior (AP) and oblique views showing no presence of a foreign body.

Clinical examination revealed a 1 cm x 1 cm puncture wound with swelling on the radiopalmar aspect of the right hand (Figure 2). The right thumb exhibited tenderness upon motion; however, the hand compartments were soft and well-perfused, with a capillary refill time of less than 2 seconds and intact sensation to ensure there was no ongoing compartment syndrome of the hand. Radiographs of the right hand showed no evidence of fracture or retained foreign bodies (Figure 3).

The patient received 0.5 mL of tetanus toxoid intramuscularly. For pain relief, 100 mcg of intravenous fentanyl was administered, reducing his pain score to 2. The initial wound care involved thorough irrigation with copious saline. Empirical broad-spectrum intravenous ciprofloxacin was started to cover potential marine pathogens. Due to the puncture wound, formal debridement of the right hand was scheduled on the day of presentation.

Under general anesthesia, surgical exploration revealed an injury to the deep thenar muscle accompanied by 2 cc of hematoma. No foreign bodies were found, so the wound was left open and packed with povidone-soaked ribbon gauze [3]. Daily wound inspections and monitoring were performed. The patient was discharged on postoperative day 3 with instructions to complete a one-week course of antibiotics. One month after surgery, the wound had completely healed, full functionality of the thumb and right hand was restored, and the patient had resumed normal daily activities.

## Discussion

Stingrays are cartilaginous fish, related to sharks and skates, with approximately 150 species recognized globally [3]. They are distinguished by their flattened bodies, long tails, and wing-like pectoral fins that are fused to their heads, granting them exceptional maneuverability [1-3]. In this case, the patient provided a photograph of the stingray at the emergency department, allowing clinicians to accurately identify the species and tailor management strategies based on variations in venom composition and associated risks.

The species identified is a freshwater stingray belonging to the family Potamotrygonidae, specifically *Potamotrygon leopoldi*. This stingray is recognized by its distinctive spotted or mottled pattern, featuring contrasting pale spots on its body.

Stingray injuries usually occur from accidental encounters during activities such as wading, swimming, or fishing [1]. The venomous spines on a stingray’s tail can inflict puncture wounds, lacerations, and envenomation. In some cases, fragments of the barb may break off and remain as foreign bodies within the wound [2]. Additionally, the venom often produces intense, localized pain, which is why many patients present to the emergency department with severe, excruciating pain at the injury site [1-4].

Trickett et al. have outlined an effective management algorithm for these injuries. The primary first aid measure is thorough wound irrigation to remove venom and debris [3]. Immersing the affected area in hot water at a temperature as high as can be comfortably tolerated by the contralateral hand provides significant pain relief. Since stingray venom is heat-labile, immersion in hot saline or freshwater at 43°C to 46°C is recommended to denature the venom proteins [1].

Given the high risk of bacterial contamination in marine environments, prophylactic antibiotics are advised for patients with stingray injuries [1,5]. A study by Myatt et al. showed that among 71 patients treated with prophylactic antibiotics, only one developed a secondary wound infection, compared to five of 30 patients who did not receive antibiotics [4]. Many experts recommend a five to seven-day course of fluoroquinolones to effectively prevent infection [5].

In summary, thorough wound irrigation and debridement are critical in managing stingray injuries. Debridement not only removes venom and debris but also helps eliminate any retained foreign bodies, such as fragments of the stingray barb, which may be difficult to detect on radiographs due to their non-radiopaque nature [3,5]. Surgical intervention further allows for the identification and repair of any structural damage, thereby restoring function and minimizing complications. Professionals handling stingrays, such as pet shop owners, beach guards, and aquarists, should wear protective gear, receive proper training, and use safe handling techniques to prevent injury from the stingray's venomous barb.

## Conclusions

Stingray sting injuries, though rare, present unique challenges due to the combined effects of mechanical trauma, envenomation, and the risk of secondary infection. Optimal management requires a thorough understanding of the injury mechanism, prompt first aid measures such as hot water immersion, meticulous wound irrigation and a careful assessment for any retained foreign bodies or structural damage. Early administration of prophylactic antibiotics and timely surgical intervention are crucial to prevent complications and promote effective healing. This case underscores the importance of vigilance and preparedness, even beyond the stingray’s natural habitat, to minimize risks and achieve favorable patient outcomes.
